# Probiotics and oral-periodontal health

**DOI:** 10.1186/1878-5085-5-S1-A125

**Published:** 2014-02-11

**Authors:** Mahmet C Haytac, Anatoly A Kunin, Irina A Belenova

**Affiliations:** 1 Professor, Cukurova University, Faculty of Dentistry, Turkey; 2 Professors, Dr. Med. Sc., Voronezh N.N. Burdenko State Medical Academy, Therapeutic Dentistry Department, Voronezh, Russia

## 

Periodontitis is a chronic inflammatory disease culminating in loss of periodontal support structures. The etiology of this destructive disease is primarily related to biofilm formation with certain putative bacterial pathogens. The development of antibiotics resistance by certain important pathogens has increased the possibility of returning to the pre-antibiotic age. During last decade, identification of health-associated and potentially beneficial bacterial species that may reside in the gingival sulcus and oral cavity has been the topic of numerous studies focused on prevention and treatment of certain diseases. Probiotics are live microorganisms (in most cases, bacteria) that are similar to beneficial microorganisms found in the human gastrointestinal tract. They are also called “friendly bacteria” or “good bacteria”. Commonly, most of the species ascribed as having probiotic properties belong to the genera *Lactobacillus* and *Bifidobacterium*. Use of probiotics to maintain oral or periodontal health can provide a natural defense against bacteria, which are thought to be harmful to teeth and gums. Thus, identification of useful probiotics and establishing the most appropriate dose and vehicle for their use are areas of active investigation. While, dairy products have been most investigated, other means of probiotics use such as those in chewing gums or lozenges have also been studied. Although probiotics are in the initial stages of scientific research and application, they offer a natural and promising therapeutic approach. Accordingly, we will discuss latest research data on probiotics use for prevention and treatment of oral and periodontal diseases.

